# Nucleotide Exchange Mechanism Involving Angle-Dependent Rate Constants Extracted from F1-ATPase Single-Molecule Rotation Trajectories

**DOI:** 10.1021/acs.jpcb.5c04403

**Published:** 2025-11-10

**Authors:** Sándor Volkán-Kacsó, Ricardo A. Matute, Maria-Elisabeth Michel-Beyerle, Oganes Khatchikian, Rudolph A. Marcus

**Affiliations:** †Noyes Laboratory of Chemical Physics, #Marcus Center for Theoretical Chemistry, 6469 California Institute of Technology, 1200 E. California Blvd., Pasadena, California 91125, United States; ‡ Segerstrom Science Center, Azusa Pacific University, 901 E. Alosta Ave., Azusa, California 91702, United States; § Departamento de Química, 28090 Universidad Técnica Federico Santa María, Avenida España 1680, Valparaíso 2340000, Chile; ∥ 9184 Technical University of Munich, Arcisstr. 21, 80333 Munich, Germany

## Abstract

Evidence has been mounting that in the rotational cycle of F1-ATPase there is a concerted ATP binding and ADP release that yields a million-fold acceleration in the rate of the product ADP release. We developed a theory of reaction kinetics to investigate the relationship between the concerted nucleotide exchange and previous single-molecule forced rotation data from AdachiK.,  Nat. Commun. 2012, 3, 1022 22929779 10.1038/ncomms2026PMC3449090. We extracted from these data angle-dependent rate constants for nucleotide binding and release. The rate constants were then used in a unified kinetic scheme, also consistent with other single-molecule and ensemble experiments, to obtain analytical equations for nucleotide occupancy change events from nano- to millimolar ATP concentrations. A theory-experiment comparison revealed novel evidence about the concerted mechanism: it is determined by correlated conformational changes in the F1-ATPase ring, and its kinetic signature is a unified angle-dependent function of the nucleotide binding and release rate constants, which is independent of ATP concentration.

## Graphical abstract

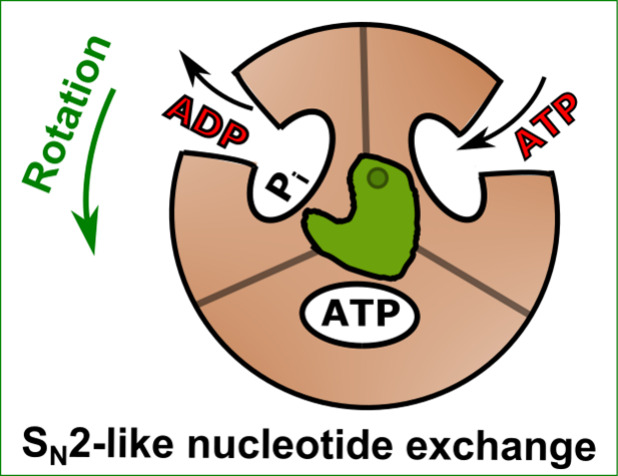

## I Introduction

For more than a century, the Michaelis–Menten (MM) equation for treating enzymes has been and continues to be very useful.^1^ The MM equation does not uncover the details that were subsequently found using X-ray crystallography^2^ and single molecule structure^3^ and imaging^4^ studies of various types of enzymes. The F1-ATPase biological motor enzyme was shown in both single-molecule and ensemble experiment to obey the MM equation.^5,6^ This biological nanomotor, while being a minimal working subcomplex of the ATP synthase, is itself a complex enzyme: its α_3_β_3_ ring segment contains three active binding sites^2^ in which mechano-catalysis occurs that drives the rotation of a central γ shaft.

The rotation proceeds in discrete steps that occur at random times.^5,7^ Focusing on individual steps with a ‘divide and conquer’ approach, single-molecule imaging of free and forced rotation has revealed that these steps correspond to elementary processes in the rotary catalysis of ATP: the binding of reactant nucleotides to the F1-ATPase, its hydrolysis in the reaction pocket, and the release of products, ADP and Inorganic Phosphate.^7^ Notably, coordinated nucleotide exchange was discovered using high-speed imaging by Kinoshita and Noji^5^ who suggested that, in the active rotational kinetics of F1-ATPase, binding of a nucleotide to one subunit proceeds in the same substep with the release of another nucleotide from another subunit.^8^ The concerted behavior was later confirmed^7,9^ by further experiments on *Thermophilic Bacillus* F1-ATPase. In a previous theoretical work,^10^ a role for the concerted behavior was suggested to cause an increased rate of ADP release by a factor of at least 10^5^ compared to its release unaided by ATP binding. Otherwise, this spontaneous ADP release would be a bottleneck process with a lifetime in the range of seconds.

The complete ATP synthase driven by a proton gradient across a membrane rotates in the opposite direction of F1-ATPase in order to achieve its function of ATP synthesis from ADP and inorganic phosphate. Microscopic reversibility requires that the ‘elementary’ kinetic substeps, resolved for F1-ATPase, would be reversed in ATP synthase. Accordingly, ATP synthase presumably uses the concerted nucleotide exchange in reverse compared to F1-ATPase. So, in an up-to-date picture of rotational ATP synthesis, concerted nucleotide exchange also coordinated with the γ shaft rotation is proposed, instead of a sequential mechanism.^1^ In the new picture, a fast nucleotide release permits a high turnover rate of a thousand ATP molecules synthesized every second by an efficient rotary synthesis of life’s primary fuel molecule. However, the relation between rotational kinetics and the allosteric interaction between the two binding sites involved in the concerted nucleotide exchange is not yet understood. Its elucidation has the potential to advance medical^11–13^ and biomedical research.^14,15^ A goal of this work is to study the concerted process using a theoretical treatment of controlled rotation experiments by extracting and using angle-dependent rate constants. Specifically, the question asked in this paper is a conditional one, namely, given that an ATP that was bound goes on to hydrolyze, what can one infer about the angle dependence of the rate constant? We found that in the reaction kinetics there is a characteristic unified angle-dependent functional form for the nucleotide binding and release rates, which implies concerted conformational changes in the enzyme at all concentrations and nucleotide occupancies. By extending the kinetic model to all ATP concentrations, this work complements a previous treatment of lifetime distributions of long nucleotide binding events.^16^ It addresses a problem in biophysics of developing methods to extract physiologically relevant kinetic quantities from force spectroscopy trajectories of single biological macromolecules. The problem is challenging because the external forces applied in these systems driving them out of equilibrium.^17–19^ By applying the theory to experimental data from individual specimens engineered for single-molecule imaging, we demonstrate an effective quantitative methodology to gain mechanistic insight into the nucleotide exchange which enables the high turnover rate of the ATP synthase.

An outline of this article is as follows: In Unified Theory of Angle-Dependent Kinetics at All ATP Concentrations, we describe a unified kinetic treatment of the ATP hydrolysis cycle in F1-ATPase intended to cover a wide range of ATP concentrations, both physiological and subphysiological. We derive an analytical expression for the population of nucleotide change events, including the physiologically relevant millimolar range. It uses a functional form extracted from experiments using a method described in the following section. In Method to Extract Angle-Dependent Rate Constants from Controlled Rotation Experiments, we describe a statistical method for extracting the angle-dependent populations and rate constants from previously published experimental data. The procedure, unlike earlier calculations that used angle-independent lifetimes, does not assume a particular functional form for the rates or dwell time distributions. Instead, the functional form is extracted from experiments. In Section 3, a theory-experiment comparison of angle-dependent nucleotide populations is performed at low ATP concentrations, where data are available. The emergence of concerted behavior is demonstrated as the nucleotide concentration is increased from nanomolar to micromolar range. In Section 4, we discuss the evidence that the angle-dependent rate binding and release rate constants extracted at low ATP concentrations are relevant in the physiological range. We conclude in Section 4 with a summary of the principal findings.

## II Theoretical and Data Analysis Methods

### Unified Theory of Angle-Dependent Kinetics at All ATP Concentrations

In this section, we describe a theory of kinetic events in controlled rotation experiments. The rotational kinetics in F1-ATPase was shown to follow a well-defined periodic sequence of kinetic states.^7^ In single-molecule imaging trajectories, a signature of the sequential kinetics is indicated by a periodic sequence of steps and substeps separated by dwells of stochastic duration.^5^ The observation of substeps presents an opportunity to study the motor enzyme by a ‘divide and conquer’ approach that focuses on individual kinetic substeps,^5,8^ which resulted in a detailed scheme for F1-ATPase kinetics coupled with rotation.

Here we extend this scheme of the physiologically relevant millimolar ATP concentration to include both low (nanomolar) and intermediate (micromolar) ATP concentrations. In Figure 1, both low and high ATP concentration regimes are shown. The kinetics is applied to events detected as steps in the angular position of the γ shaft monitored in single-molecule experiments. Arguably, at the time scales resolved by these experiments, the transitions between kinetic states of the motor enzyme follow a rate kinetics described as angle-dependent rate constants.^20^ In a kinetic state, the conformation of the ring subunits and the monitored rotation angle undergo fluctuations in the vicinity of a potential minimum. The angle-dependent rate constants can vary by orders of magnitude as functions of the rotation angle θ. A notable exception is the synthesis rate constant, which was found to be independent of the rotation angle.^8^

**Figure 1:**
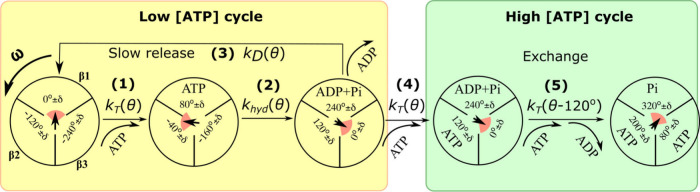
Extended kinetic scheme for F1-ATPase nucleotide binding and release events monitored during enforced rotation with angular velocity ω. Parenthetic numbers (1) – (5) on the diagram indicate reaction kinetic steps that correspond to eqs 1–5. Also shown are the angle-dependent rate constants from eqs 1–5 for the kinetic steps represented by horizontal arrows. A binding or release of a nucleotide is indicated by curved arrows. Both low and high ATP concentration regimes are shown with nucleotide occupancy levels ranging from 0 to 2. For each of the kinetic states, the angular position of the γ shaft is given explicitly, and it is also indicated by an arrowhead, and a range of angles δ ≈ 40° in which various steps occur is represented by a red circular slice. The low [ATP] cycle (yellow highlight) is characterized by slow ADP release and the equilibrium condition in eq 6; the high [ATP] cycle (green highlight) is characterized by a concerted ATP binding and ADP release.

It is customary to represent the kinetic states in F1-ATPase by indicating the occupancy of each binding site, the likely position of the rotary shaft, and the conformational state at the potential minimum configuration. This type of representation is seen in the diagrams in Figure 1; the F1-ATPase is represented as a circle divided into three “slices”, each representing one active binding site and a central asymmetric shape representing the angular position of the rotatory shaft.

For notational simplicity, we use a reduced notation reminiscent of standard chemical reactions. Denoting the enzyme without any bound nucleotides by *E*, an ATP molecule by *T*, an ADP by *D*, and an Inorganic Phosphate by *P*_*i*__,_ a 5-step kinetic scheme is given in eqs 1–5. A corresponding extended notation is given in Figure 1.

1 $$T+E\left(\theta_{1}\right)\overset{k_{T}\left(\theta_{1}\right)}{\to }ET\left(\theta_{1}\right)$$

2 $$ET\left(\theta_{1}\right)\overset{k_{h}\left(\theta_{1}\right)}{\to }EDP_{i}\left(\theta_{1}\right)$$

3 $$EDP_{i}\left(\theta_{3}\right)\overset{k_{D}\left(\theta_{3}\right)}{\to }E\left(\theta_{3}\right)+D+P_{i}$$

4 $$T+ED\left(\theta_{4}\right)\overset{k_{T}\left(\theta_{4}\right)}{\to }EDT\left(\theta_{4}\right)$$

5 $$T^{\prime }+EDT\left(\theta_{5}\right)\overset{k_{T}\left(\theta_{5}+240^{{}^{\circ}}\right)}{\to }ET^{\prime }T\left(\theta_{5}+240^{{}^{\circ}}\right)+D$$

Eqs 1–5 represent a set of equations that describe controlled rotation experimental data that involve reactant nucleotide binding and subsequent product nucleotide release, over a wide range of ATP concentrations. At low ATP concentrations (nano- to micromolar), F1-ATPase undergoes a slow spontaneous ADP release process (eq 3) with angle-dependent rate constant *k*_*D*_(θ_3_). Leading up to this slow release reaction, steps 1 and 2 (eqs 1 and 2) occur in quick succession at the same rotation angle, so θ_2_ = θ_1_. Due to a lack of correlation in step 3, we introduce a new angle, θ_3_. Together, steps 1–3 describe the low ATP concentration cycle (Figure 1, yellow highlight). Because of the rapid disappearance of *ET*(θ_1_) in step 2, we set (*d*/*dt*)*ET*(θ_1_) ≅ 0, the steady-state approximation. We have

6 $$k_{hyd}\left[ET\left(\theta_{1}\right)\right]=k_{T}\left[T\right]\left[E\left(\theta_{1}\right)\right]$$

To further simplify the notation in eqs 4–5 we absorbed the *P*_*i*_ into the *ADP*, so *D* can denote *ADP* or *ADP* and *P*_*i*_ in the same pocket. The release of *P*_*i*_ can occur as a separate step, and it is undetectable in the controlled rotation experiments. At micro- to nanomolar ATP concentrations, ATP hydrolysis is followed by slow “unisite” ADP release (cf. Figure 1, yellow highlight). At such low ATP concentrations, the reactions tend to occur at different uncorrelated angles; therefore, θ_3_, θ_4_, and θ_5_ are treated as separate, independent variables.

At the higher (micro- to millimolar) ATP concentrations, the kinetics follows the step in eq 4, where there is a multiple occupancy of E (cf. scheme from Figure 1). So, the product state of eq 2 goes on to gain another nucleotide in eq 4, and there is also an alternative route in eq 3. This spontaneous and slow nucleotide release was reported by Senior^21^ and confirmed by Kinoshita and co-workers.^9^

Turning to the high ATP concentration regime, in eq 5 the θ_5_ denotes the rotation angle as defined relative to the site occupied by the first nucleotide after *‘E’*, i.e., θ_5_ is the rotation angle define relative to the “ejector” subunit occupied by *D*. The primed quantity *T′* denotes the angle of the nucleotide “acceptor” subunit (β_3_ in Scheme 1). A strong binding-release cooperation implies that the (fast) ADP release rate must tend to the binding rate *k*_*T*_(θ) in the ejector β subunit; hence, the 240° shift (cf., eq 6).

When both slow and fast ADP release mechanisms are present, we unite the net ADP release rate as a sum of the spontaneous and fast ADP release rates,

7 $$k_{D}^{tot}=k_{D}+k_{T}$$

For ATP binding, we assume a collision-like process,^22^ and so, the ATP binding rate constant is written in eqs 9–10 as a product of a bimolecular rate constant *k*_*T*__0_, the ATP concentration [ATP], and an angle-dependent ‘structure’ function *g*(θ). The latter is determined by the conformational constraints in the F1-ATPase structure, a connection which we articulate in the Discussion section. We postulate, based upon experimentally determined angle-dependent rate constants described in the Results section, that the same structure function *g*(θ) is also used for the *k*_*D*_, when shifted by 240°.

8 $$k_{T}\left(\theta \right)=k_{T0}\left[\mathrm{ATP}\right]g\left(\theta \right)$$

9 $$k_{D}\left(\theta +240^{◦}\right)=k_{D0}g\left(\theta \right)$$

Using the conditions from eqs 8–9, we consider a steady rotation rate, as in controlled rotation enforced by magnetic tweezers,^23^ θ = *ωt*. So, the rotation angle is proportional to time, yielding kinetic equations similar to those introduced in a previous article.^16^ These equations given in the SI as eqs S3 and S6 differ from standard chemical kinetic equations because of their time-dependent ‘rate constants’. We denote the angle-dependent populations of empty states that accept an ATP as *P*_*E*_(θ). These states correspond to the reactant states (left side) in eqs 1, 3, and 5. We denote the population of ADP bound states undergoing ADP release as *P*_*D*_(θ). These states then correspond to the product states (right-hand side) of eqs 2, 3, and 5. Then, using the rate constants in eqs 1–5, the populations of states *P*_*E*_(θ) and *P*_*D*_(θ) can be expressed by solving eqs S7 and S10 (cf., the SI Methods) as,

10 $$\ln P_{E}\left(\theta \right)=-\frac{k_{T0}\left[\mathrm{ATP}\right]}{\omega }\int_{\theta_{0}}^{\theta }g\left(\theta^{\prime }\right)d\theta^{\prime }$$

11 $$\ln P_{D}\left(\theta +240^{◦}\right)=-\frac{k_{D0}+k_{T0}\left[\mathrm{ATP}\right]}{\omega }\int_{\theta_{0}}^{\theta }g\left(\theta^{\prime }\right)d\theta^{\prime }$$

In eqs 10–11, the lower limit of the integration θ_0_ is set at a reference angle where the integrand is negligible. With this definition, the results of the integrations are independent of θ_0_. *g*(θ) shows a characteristic “volcano” shape, described in the Results section, and so, the value of θ_0_ is set at its “base”. As such, θ_0_ is not the initial angle in the controlled rotation experiments. We note that eqs 10–11 describe the angle-dependent populations of *P*_*E*_(θ) and *P*_*D*_(θ) in a wide ATP concentration range extending from the nanomolar to micromolar.

### Method to Extract Angle-Dependent Rate Constants from Controlled Rotation Experiments

In the previous section, we provided a theory of angle-dependent kinetics. In this section, we present a method to analyze data from controlled rotation experiments performed by Adachi et al.,^23^ experiments which probe the rotation of single F1-ATPase motor enzymes while also monitoring the individual binding and release of ATP and ADP molecules. These events shown in Figure 2A are detected as single nucleotide occupancy changes of the enzyme as a function of the rotation angle. Pairs of events (cf. Figure 2B) of nucleotide increase by one (binding) and subsequent decrease by one (release) are used to count angle-dependent populations (Figure 2C) which then can be compared with theory.

**Figure 2:**
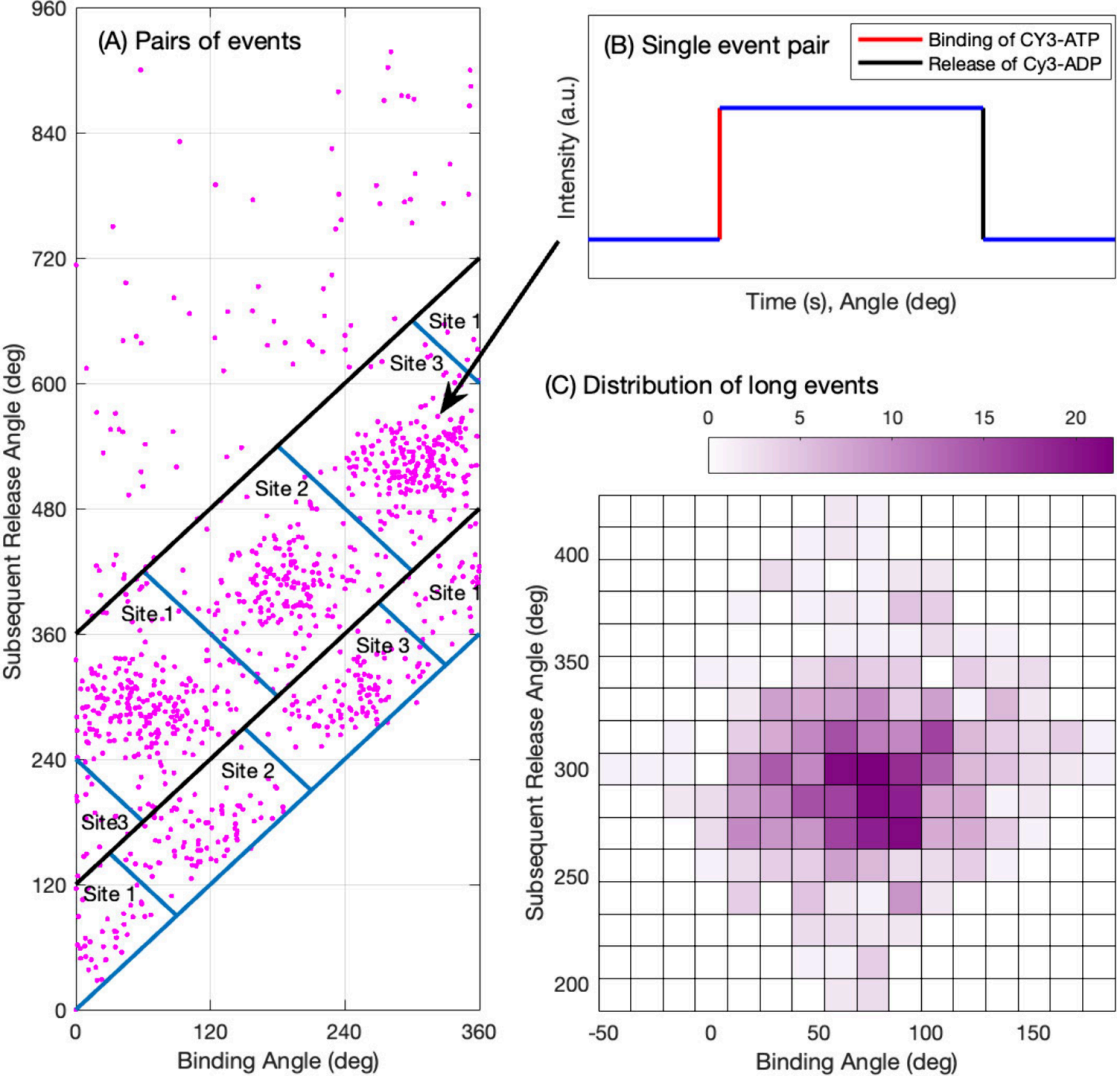
ATP binding and ADP release event pairs in single-molecule trajectories from data published by Adachi et al.^5^ (A) Scatter plot of binding and subsequent release angles for fluorescent Cy3-ATP detected in controlled rotation experiments, adapted from a figure in Adachi at al.^5^ (B) Each dot represents a pair of binding and subsequent release events. Data from single-molecule trajectories at 25 nM ATP concentration are shown. (C) Discrete probability distribution function approximated as a 2-dimensional histogram calculated from the long binding events from (A). The data points were placed in angular bins of 15° defined by their binding and their subsequent release angles. The 3 sites were assumed to be statistically identical, which allowed shifting the angles of the points assigned to the second and third subunits by 120° and 240°, respectively. This operation resulted in larger event counts in each bin, as indicated by the color chart.

When the γ shaft is rotated by an external torque via magnetic tweezers,^10,13^ the dwell time distribution of binding and release events (distribution of times for ATP to bind and product ADP to release) is observed to be multiexponential.^14^ For long dwells during which the associated rates have changed significantly with the angle, an internally consistent method for extracting rate constants and populations requires a procedure without the assumption of angle-independent rates.

The method applies when the angle of rotation is driven at a constant rate ω. In each cycle of rotation, a new member of a population of these events is added or subtracted with some probability and the multiplicity of cycles is treated as an ensemble. To ensure an extraction of kinetic quantities that is unbiased and self-consistent, in the sense that kinetic rates are not a priori assumed to be constant, we use the following three-step approach:

#### Site Assignment

In controlled rotation experiments, the individual nucleotide binding and release events are monitored using single-molecule fluorescence.^13^ The experiment does not distinguish between the binding sites of the Cy3 fluorophore-tagged ATPs or ADPs. So, it is necessary to assign binding and release events to one of the three active subunits. Based upon previous observations^10^ the binding and release of nucleotides depends strongly on the rotation angle. Effectively, the rates of binding and release peak in a certain angular range; therefore, the ATP binding and subsequent release events (of ADP following hydrolysis) tend to occur in angular clusters around these peaks, seen in Figure 2. Then, following the procedure described by Adachi et al.,^10^ we identify the subunits with the clusters of events.

#### Angular Binning of Nucleotide Binding and Release Event Populations

To extract nucleotide binding and release rate constants, we introduce two states, state “0” and state “1”. State “0” denotes an empty state (no nucleotide bound to a binding site of interest), and state “1” denotes a bound state (there is a nucleotide bound to the site of interest). We note that the method is applicable when multiple kinetic events occur between transitions from 0 to 1 or 1 to 0, events that do not involve nucleotide binding or release. As such, a 0 → 1 event can be ATP binding, and 1 → 0 can be the ADP release following a hydrolysis event (undetectable).

Transitions from state 0 to state 1 occur according to an angle-dependent (and so time-dependent) gain rate *R*_*gain*_(θ). The population of state 0 can also increase by a competing process described by the loss rate *R*_*gain*_(θ). To extract the angle-dependent population gains and losses, the events are placed in angular bins (θ – *Δθ*/2, θ + Δθ/2) of size Δθ centered at angle θ as seen in Figure 2C. The Δθ is the bin size and is distinct from *dθ*, which is an infinitesimal quantity that is involved in the differential equations and integrals derived from the reaction kinetics. In enforced rotation with constant rate ω, the angular range of a bin is scanned in time Δ*t* = Δθ/ω. The binned event counts yield the number gain (0 → 1) and loss (1 → 0) events, Δ*N*_0→1_ (θ) and Δ*N*_1→0_(θ). If the angular bin size is sufficiently small so that the gain and loss rates do not change significantly within the range of a bin, the rates are approximated as

12 $$R_{gain}\left(\theta \right)=\frac{\Delta N_{0\to 1}\left(\theta \right)}{\Delta \theta /\omega },R_{loss}\left(\theta \right)=\frac{\Delta N_{1\to 0}\left(\theta \right)}{\Delta \theta /\omega }$$

#### Extracting Angle-Dependent Rate Constants

In the time interval Δ*t* = Δθ/ω, transitions from state 0 to state 1 and back follow angle-dependent (and so time-dependent) rates described by gain-loss equation using angle-dependent gain *R*_*gain*_ (θ) and loss *R*_*gain*_ (θ) rates from eq 12. For sufficiently small Δ*t*, the population associated with state 0 evolves according to difference equation

13 $$\frac{\Delta N_{0}\left(\theta \right)}{\Delta t}\approx R_{gain}\left(\theta \right)-R_{loss}\left(\theta \right)$$

The rate constants for the loss (forward) processes can be then extracted from the loss rate, by dividing with the population *N*_0_(θ) of cycles with 0 state at angle θ. For ADP release, we find,

14 $$k_{D}\left(\theta \right)=\frac{R_{loss}\left(\theta \right)}{N_{0}\left(\theta \right)}\approx \frac{\omega \Delta N_{1\to 0}\left(\theta \right)}{N_{0}\left(\theta \right)\Delta \theta }$$

The total number of cycles in a trajectory, *N*_*tot*_, yields the combined population of 0 and 1 states. The rate constant for the gain (reverse) term can be extracted from the loss term, but the population of the 1 state individuals *N*_1_(θ) = *N*_*long*_ – *N*_0_(θ) must be known at angle θ. So, for ATP binding,

15 $$k_{T}\left(\theta \right)=\frac{R_{gain}\left(\theta \right)}{N_{1}\left(\theta \right)}\approx \frac{\omega \Delta N_{0\to 1}\left(\theta \right)}{\left[N_{tot}-N_{0}\left(\theta \right)\right]\Delta \theta }$$

We note that extracting the angle-dependent quantities from rotation driven at a constant rate is analogous to a method used for treating force-ramp experiments in protein folding.^17–19^

## III Results: Comparison of Theory and Controlled Rotation Experiments

In Figure 3 we have plotted the angle-dependent rate constants (dots) and associated confidence intervals (vertical bars) extracted using the procedure described in Method to Extract Angle-Dependent Rate Constants from Controlled Rotation Experiments from the data of Adachi et al. plotted in Figure 3. These data are consistent with earlier stalling experiments.^8^ We follow the convention by Kinosita and Noji^7,23,24^ and define the origin for the rotation angle θ = 0 when the system is in the dwell preceding the binding of an ATP molecule to a chosen reference subunit β_1_. These angle-dependent trends are similar to a previous analysis,^8^ but the plots on Figure 3 are quantitatively more accurate because they were extracted using an unbiased method, without assuming a functional form for their angle independence.

**Figure 3:**
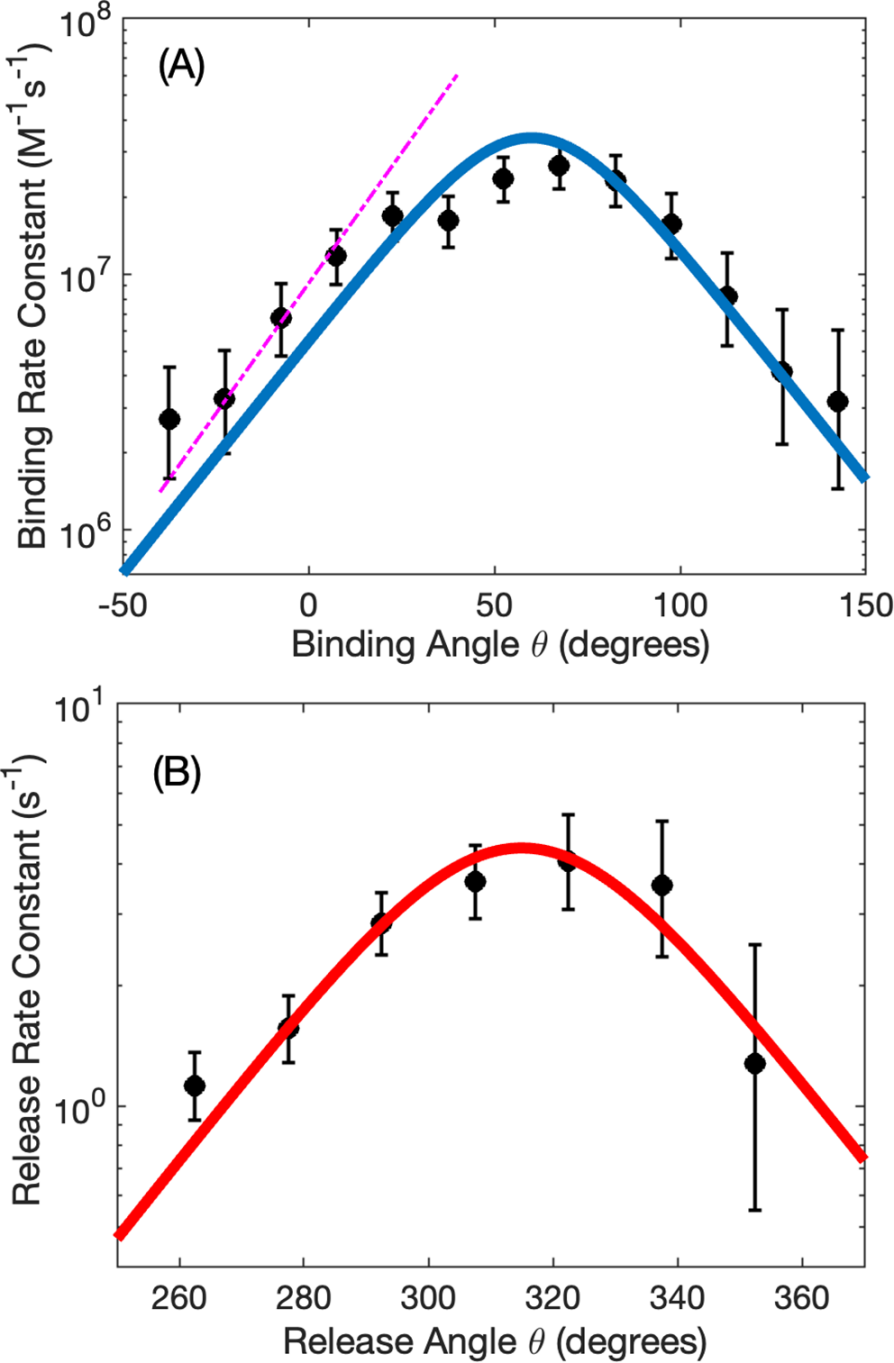
A. Bimolecular binding rate constants as a function of rotation angle for fluorescent Cy3-ATP, extracted from controlled rotation experiments (circles). Data from these single-molecule trajectories at 10 nM and 25 nM concentrations were combined. Error bars correspond to confidence levels of 95%. For comparison, the bimolecular rate constants of ATP from stalling experiments^24^ measured at 60 nM concentration are also shown as a dashed line. B. Release rate constants as a function of rotation angle for fluorescently labeled ADP (Cy3-ADP), extracted from controlled rotation experiments (circles). Data from single-molecule trajectories at 10 nM and 25 nM were combined. Error bars correspond to confidence levels of 95%.

The rate constants are seen in Figure 3 to produce distinct maxima, one per 360° cycle. These peaks display a characteristic symmetrical “volcano” shape, with their top at “turnover” angle *θ*_*TO*_ flanked by exponential slopes. A common “structure function” in eqs 8–9 was introduced for the rate constants for both nucleotide binding (Figure 2a) and release (Figure 3b), noting the 240° shift between them. To represent the experimental angle-dependent rate constants of binding and release, we introduced on Figure 3 (continuous lines) a functional form *g*(θ) = 1/[e^a(θ–2*θ*_*TO*_ + θ_0_)^ + *e* ^–*a*(θ‑θ_0_)^]. At present, we regard this form as a very interesting empirical result which can be explored further. For the binding rate constant (Figure 3A), the fitting yielded a turnover angle of *θ*_*TO*_ = 70° and reference angle θ_0_ = 50°. The latter is a value taken for convenience at the onset of binding during rotation. For the release rate constant (Figure 3B) the corresponding values are *θ*_*TO*_ = 310° and reference angle θ_0_ = 190°.

We note that the exponential dependence of the slope was observed in stalling experiments^8,24^ and treated theoretically using a model of molecular transfer.^6^ When using the present unbiased method for extracting rate constants, one finds that the angle-dependent ATP binding rate shows consistency between the controlled rotation and stalling experiments. The latter is plotted as a dashed line on Figure 3A and overlaps with the left exponential flange of the volcano plot. The same value of the exponential coefficient *a* = 0.04 *deg* ^–1^ is used in the continuous curves on Figure 3A and 3B. It was previously treated in theory^22^ and extracted from stalling experiments^8^ for the monotonically increasing side of the volcano plot. Further investigation can uncover the nature of the decreasing side of the plot. This value *a*, while consistent with the controlled rotation data on Figure 3, is not considered a parameter arising from fitting to this data.

On Figure 4, we perform a theory-experiment comparison of binding and release event populations. We plotted the experimental populations of empty states before ATP binding (dots and stars) and the populations of ADP bound states (diamonds). A direct comparison of these populations is done by rescaling the populations to unity and shifting the empty populations by 240°. We denote the normalized populations of cycles with no bound nucleotide at angle θ by *p*_*E*_(θ) and those with a bound nucleotide by *p*_*D*_(θ). They are calculated from the unnormalized populations, *P*_*E*_(θ) and *P*_*D*_(θ) following eqs S1 and S2 described in the SI.

**Figure 4:**
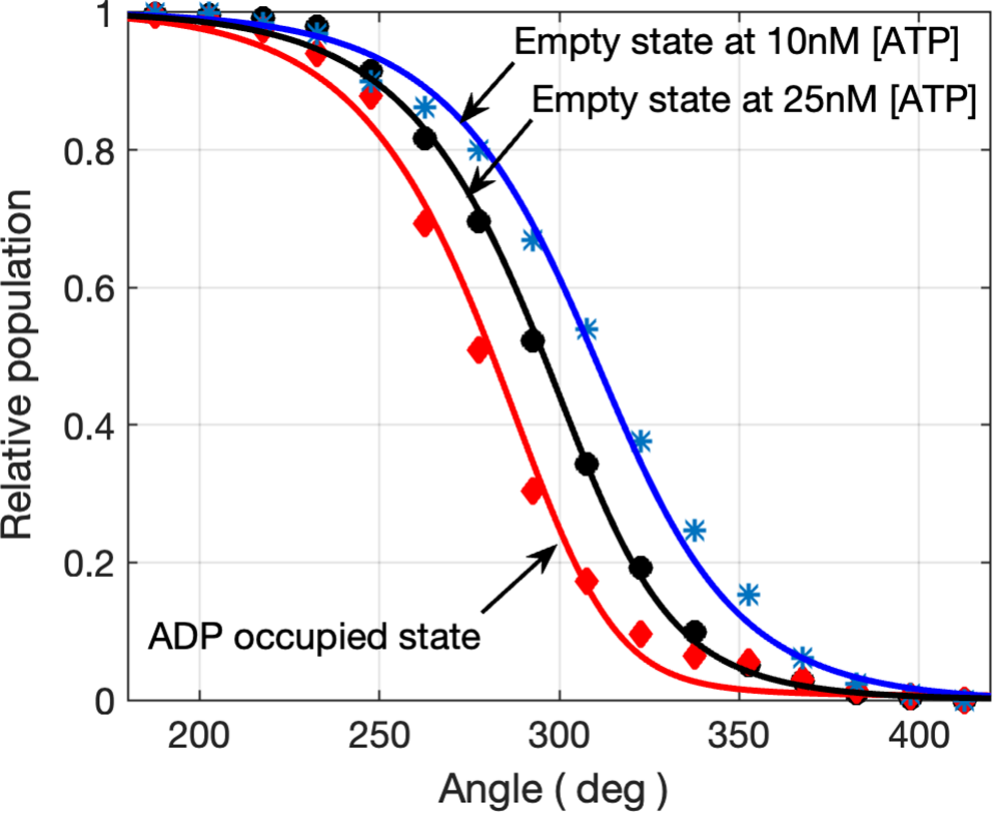
Comparison of populations of F1-ATPase with nucleotide occupancies 1 (red diamonds) and 0 (blue stars and black dots) normalized to unity. Symbols show experimental values, and solid lines show theoretical calculations using eqs 11–12 with no adjustable parameters. The red diamonds indicate the F1-ATPase ‘population’ with bound ADP decaying as a function of rotation angle. The dots and stars indicate the empty F1-ATPase populations decaying by undergoing ATP binding, at 25 nM and 10 nM ATP concentration, respectively, as a function of rotation angle shifted by 240°.

The theoretical results for the normalized populations *p*_*E*_(θ) and *p*_*D*_(θ) were calculated from eqs 10–11 using *g*(θ), boundaries, and rate constants given in an earlier paragraph in the current section. They are plotted as solid lines in Figure 4: their agreement with experiment is evident. We note that these angle-dependent populations experience a full decay within an angular range of about 80°. During an active rotation of the γ shaft, for an effective function, the decay must be complete during the up-going phase of the volcano plots from Figure 3. Any events not completed before reaching the decreasing slope have a decreased likelihood of occurring, so they may result in a rotation cycle with incomplete catalysis, which amounts to a slippage in the motor.

At higher ATP concentrations, the distribution of the binding-release event pairs on Figure 2C is predicted to gradually reduce to a line. At sufficient high ATP concentrations, this line is predicted to be shifted so that it becomes identical to a diagonal line. The effect of shifting is also seen on the angle-dependent populations in eqs 10–11 and the corresponding experimental curves in Figure 4. It is due to an increased number of nucleotides undergoing the exchange mechanism (cf., fast ADP release), events that, according to eq 7, compete with the unisite mechanism (cf., slow spontaneous ADP release). The former increases with ATP concentration (cf., eq 8) and becomes dominant at physiological concentrations, thereby guaranteeing the simultaneous nature of the binding and release, which is predicted to manifest as a complete overlap of the empty ATP binding populations with the occupied ADP releasing populations plotted on Figure 4.

## IV Discussion

### Unified Kinetics from Nano- to Millimolar Concentrations

To treat the rotation of F1-ATPase at all ATP concentrations, with an emphasis on the physiologically relevant millimolar concentration, we combined three kinetic regimes. Doing so in a unified scheme allows testing of theoretical predictions using data available from controlled rotation experiments performed at low (0 to 1) nucleotide occupancy. Eqs 1–5 represent the minimal set of forward steps that span the nano- to millimolar ranges of ATP concentration. For the analysis of reactant nucleotide (ATP) binding and subsequent product nucleotide (ADP) release events following hydrolysis, reverse processes were neglected in eqs 10–11. For the analysis of reversed rotation involving ATP synthesis, the kinetic scheme should be reversed.

In eqs 1, 3 and 5 the same binding rate constant is postulated. The rate of binding to an empty subunit is not affected by the number of nucleotides already present in the other two sites, clearly indicated by an uninterrupted linear trend in the MM curve into the nanomolar ATP concentration range [cf. ref 6]. In other words, the rate of binding to an empty subunit is not affected by the presence of nucleotides in the other two binding sites.

We propose that a similar simplifying condition applies for the hydrolysis and slow ADP release steps. At “intermediate” ATP concentrations (high nanomolar to low micromolar), following step 4 (eq 4), a hydrolysis of the ATP can occur followed by slow ADP release. The rates of these processes are similar to those of steps 2 and 3, which assumes that the presence of a stable ATP in the third binding site has little effect on these processes. Because of this similarity, the derivation of eqs 10 and 11 is unaffected and effectively accounts for these intermediate concentration events. This is verified by an inspection of Figure 1, in which the kinetics of ATP binding in process (1) and ADP release in processes 3 and 4 are independent of the ATP in subunit β_2_.

### The Shape of the *g*(θ) Function, Rate of Rotation, and Efficiency

The volcano shape of the *g*(θ) function in Figure 3 can be described in terms of an ascending phase, a turnover phase, and a descending phase (negative slope). This description implies a directional rotation of the shaft: when the rotation is in the hydrolysis direction, like in the data analyzed in this paper, the ascending phase has positive slope and is situated to the left of the turnover. During rotation, if the turnover is reached, the decreasing rate constant beyond the turnover would result in missed events in the cycle, either no binding event or no release event. These missed events are detrimental to the efficiency of the motor, so we argue that the efficiently functioning enzyme only samples the ascending phase of the volcano, never reaching the top or the descending phase.

Yet, to explore the full angular range in the controlled rotation experiments, the rate of rotation must be higher than the “natural” rate of an efficiently functioning system. If the rate of rotation is similar to the highest rate constants, found at the turnover of the volcano plot in Figure 3, the system is forced to sample angular regions likely not reached in the functioning enzyme.

### The ‘Structure’ Function and Its Implications for the Nucleotide Exchange Mechanism

A key assertion in this paper is that the *g*(θ) function is independent of ATP concentration is supported by the overlap of controlled rotation and stalling experiment binding data on Figure 3A. Stalling experiments^8^ support in part this assertion. They produced identical (within experimental error) slopes for the up-going phase of the volcano plot (dashed lines in Figure 3A), even though they were performed at higher ATP concentration using a slow-hydrolyzing mutant and so at a higher nucleotide occupancy. Further support found in ATP binding rate constants available from free rotation^5,7^ and ensemble experiments^6^ at even higher ATP concentrations, albeit not angle-resolved, is also consistent with those from Figure 3A.

The identical functional form *g*(θ) of the ATP binding and ADP release rate constants in Figure 3 and similarity of the angle-dependent population of ATP-waiting states vs those waiting for ADP release 240° later in Figure 4 suggests a structural concerted behavior in the α_3_β_3_ ring. In eqs 10–11 these populations are determined by the structure function *g*(θ): ultimately, it is the latter that kinetically “encodes” the concerted conformational behavior. A line of arguments that connects the angle-dependent features of *g*(θ) to nucleotide exchange is as follows:

We have argued that for efficiency purposes, only the ascending phase of the *g*(θ) is to be considered, both for binding and release of the nucleotides. In previous studies we proposed that binding at angles in the positive exponential slope of the binding rate constant (about 0°) is coordinated with the closing of the channel leading to the binding pocket in the β subunit.^16,22^ Applying the same logic to ADP release 240° later at angles in the ascending phase (positive slope) of *g*(θ), we must infer that the release rate constant is coordinated with the opening of the channel. There is an identical functional form *g*(θ) for a nucleotide binding and its release 240° later in Figure 2. Structurally, this means that the subunit closes about 0° and opens 240° later.

The above structural implication of *g*(θ) was made from the analysis of low ATP concentration kinetics when there is a single nucleotide undergoing binding, hydrolysis, and, later, a slow release. (Meanwhile, the other two sites are empty.) Now we consider two sites and use step 5 in Figure 1 as a concrete example. The 240° shift to a later angle in one binding site (β_3_ in Figure 1) is equivalent to no shift, i.e., complete overlap, relative to the clockwise neighbor site (β_1_, in Figure 1.). This is seen by comparing the relative angles of β_1_ and β_3_ of the reactant state in step 5 on Figure 1: Structurally, when one site (β_3_) closes as it experiences ATP binding, the second site clockwise neighbor site (β_1_) opens.

At higher ATP concentration, there is a nucleotide in the second site (β_1_), given that there was not enough time for its slow release before a nucleotide binds to the first site (β_3_). If then there is already a nucleotide in the second site (β_1_), when ATP binds to the first site (β_3_) that second site (β_1_) experiences the opening and so an immediate release of that nucleotide.

The release in step 5 then effectively has the rate constant of binding to the first site (β_3_). It means a “perfectly” coordinated nucleotide binding and release mechanism. This rate constant is proportional to the ATP concentration, so it has a range of many orders of magnitude, and at high ATP concentration, it is a fast release, orders of magnitude faster than spontaneous release.

We propose that for nucleotide exchange it is necessary that five out of six sites in the F1-ATPase ring be occupied by nucleotides (cf. the scheme of Figure 1 and eqs 1–5). There are three inactive sites permanently occupied. We speculate that the “tight” structure in the α_3_β_3_ ring constrains the nucleotide occupancy at any given time to five: so, as a sixth nucleotide is binding, the barrier for the release of the clockwise active neighbor is lowered, resulting in a concerted binding-release mechanism. So, in F1-ATPase, as an ATP transitions the binding channel in the empty subunit, the product ADP does the reverse in the releasing subunit in a concerted manner. It is expected, as in SN2 chemical reactions,^25^ that the barrier for the concerted process is dramatically lowered when compared to a sequential process.^10^ This mechanism is then the dominant mode of nucleotide ejection at physiological ATP concentrations. Computer simulations and free energy calculations using structures from recent cryo-EM experiments^26^ have the potential the reveal the constraint in the structure during the concerted nucleotide exchange that lower the barrier for ADP release. Such calculations may shed light on the upper limit for the fast ADP release, which is likely determined by the time the conformational changes propagate from the ATP binding site (β_3_ in Figure 1) to the releasing site (β_1_).

### Extracting Angle-Dependent Rate Constants in Molecular Motors

The concerted nucleotide exchange appears to be consistent with single-molecule experiments in other rotary motors, bovine mitochondrial and human mitochondrial F1-ATPases,^27,28^ suggesting a common mechanism of “auto-catalytic” nucleotide release via structural concerted allosterism.^14^

The current work offers an example of a “divide and conquer” approach to study the concerted allosterism by focusing on individual steps revealed in single-molecule observations. A key idea is the use and extraction from experimental data of angle-dependent rate constants from which then kinetic equations are built for a unified treatment of single motor mechano-chemistry.

The postulated volcano shaped structure function *g*(θ) determines these angle-dependent rate constants. Its extraction yields two effective structural parameters, the turnover angle *θ*_*TO*_ and exponential rate coefficient *a*. Focusing on the angle-dependent structure function is proposed to benefit studies of the structural origin of the allosterism in the hexameric ring^29–31^ by atomistic free energy calculations that calculate or test the parameters of the structure function.^32^ It is our extension to extend a treatment similar to the present one to other biomolecular motors, like myosin V,^33^ dynein,^34^ or the ribosome.^35^

In single-molecule free rotation experiments (i.e., those without applied external torques or constraints in the rotation), there is also information on angle-dependent rate kinetics of the rotary mechanism. Since these experiments are readily performed at physiologically relevant (high) ATP concentrations,^5,23,36,37^ developing a method for extracting angle-dependent rates from their trajectories has promising potential to test the hypothesis that the structure function *g*(θ) is independent of ATP concentration.

## V Conclusions

There is a novelty in the study, not only because angle-dependent rate constants are extracted and used for individual rotation steps instead of “pure” constants in the single-molecule experiments but also because the theory involves a unified treatment of rotation kinetics at low concentrations of the nucleotide and at high concentrations. In the former, there is more randomness in the entry and exit of the nucleotides, and in the latter, there is a strong correlation between the entry and exit in the F1-ATPase amounting to a nucleotide exchange.

The theoretical treatment given in this article describes both mechanisms and uses analytical theory to connect the two. A key component of the kinetic model of the concerted nucleotide exchange mechanism is a volcano-shaped function for angle-dependent kinetic rate constants. This ‘structure function’ is presumably the same at all ATP concentrations and nucleotide occupancies and is determined by the structural opening and closing conformations of the nucleotide binding sites in the α_3_β_3_ ring of the F1-ATPase. We have argued that the conformational changes occur in concert with nucleotide binding and release. So, by connecting these conformational changes with the angle-dependent shape of the structure function, we found that the concerted nucleotide exchange has a kinetic signature detectable in the controlled rotation data.

## Supplementary Material



## Notes

Description of normalization method used to generate Figure 4 and detailed derivations of eqs 10–11 (PDF)
