# Supplementary material for: Nucleotide Exchange Mechanism Involving Angle-Dependent Rate Constants Extracted from F1-ATPase Single-Molecule Rotation Trajectories
Source: J Phys Chem B. 2025 Nov 10;130(2):668–76. doi: 10.1021/acs.jpcb.5c04403 (PMC12814531; doi:10.1021/acs.jpcb.5c04403)
Supplement: Supplementary file 1 [file jp5c04403_si_001.pdf]

Supporting Information for article

Nucleotide Exchange Mechanism Involving Angle-dependent Rate Constants Extracted from F1-ATPase Single-molecule Rotation Trajectories

Sándor Volkán-Kacsó [a,b], Ricardo A. Matute[a,c], Maria-Elisabeth Michel-Beyerle[d], Oganes Khatchikian[a], and Rudolph A. Marcus* [a]*

[a]Noyes Laboratory of Chemical Physics, California Institute of Technology

1200 E. California Blvd., Pasadena, CA 91125, USA

[b]Segerstrom Science Center, Azusa Pacific University

901 E. Alosta Ave., Azusa, CA 91702, USA

[c]Departamento de Química, Universidad Técnica Federico Santa María,

Avenida España 1680, Valparaíso 2340000, Chile.

[d]Technical University of Munich, Arcisstr. 21, 80333 Munich, Germany

1. Normalized populations in the controlled rotation

To calculate the theoretical counterpart of $p_E(\theta)$ and $p_D(\theta)$ we use Eqs. 9-10. For binding events, we subtract the probability $P_E(\theta_c)$ that no binding occurred in the whole cycle (which were not counted in the experiment), and so the empty (unbound) state survived to the end of the `binding window' at an angle $\theta_c \approx 160^o$. The probability is then normalized by dividing with the probability of a long binding event per cycle $1 - P_E(\theta_c)$, yielding

$$p_E(\theta) = \frac{P_E(\theta) - P_E(\theta_c)}{1 - P_E(\theta_c)}. \qquad (S1)$$

To normalize the population of bound states, we proceed in analogy, with the shorthand notation $\theta'' = \theta + 240°$ is introduced for the angular shift. First, we subtract the probability $P_D(\theta_c'')$ of bound states surviving to an angle $\theta_c'' = \theta_c + 240° \approx 400^o$ beyond the closing of the `release window'. This is followed by dividing with the probability $1 - P_D(\theta_c'')$, and so

$$p_D(\theta'') = \frac{P_D(\theta'') - P_E(\theta_c'')}{1 - P_E(\theta_c'')}. \qquad (S2)$$

2. Detailed derivation of theoretical expressions for populations, given in the text, at low controlled rotation rate

To treat the rotation of F1-ATPase at all ATP concentrations, including the physiologically relevant millimolar concentration, in the model we suggest three kinetic cycles each becoming dominant in a different range of ATP concentration. One of the cycles is dominant at the physiologically relevant millimolar ATP concentration (Fig. 1, green highlight). In it the binding of ATP induces a simultaneous fast release of ADP resulting in a close to 3 nucleotide occupancy. The other two cycles are dominant at sub-millimolar to nanomolar ATP concentrations yielding 0 to 1 occupancy states and they are observed in the controlled rotation experiments. They are both characterized by a slow ADP release unaided by the binding of ATP.

We postulate the that in the physiologically relevant function of the ATP Synthase, the probability $P_E(\theta)$ that the β_1 is empty at the beginning of the cycle (at θ_0) is close to unity. It means that a nucleotide, bound to β1 in the previous cycle, has undergone hydrolysis (or synthesis, depending on the direction of artificial rotation) and the products were released. The population of empty states (β_1 subunit) decays according to the binding rate constants $k_T(\theta)$ assumed to be angle-dependent, yielding an equation

$$\omega \frac{dP_E(\theta)}{d\theta} = -k_T(\theta)P_E(\theta). \tag{S3}$$

In the current analysis we use Eq. 1 to describe the binding of an ATP that goes on to form an ADP later in the kinetic cycle ("long binding events") and not to an ATP that is released back to the solution before it could undergo hydrolysis.

Turning to the ADP release at the end of kinetic scheme on Scheme 1: let $P_T(\theta)$ denote the angle-dependent probability occupancy of subunit β1 at an angle θ by a "long time" ATP that was added at an initial angle θ_0. We define $P_D(\theta + 240°)$ as the occupancy probability of ADP 240° later. Thereby we treat in the theory only the ATPs that survived as ADP's some later and do not consider those ATP's that returned as ATP's to the solution ("short events"). ADP release it can proceed along two kinetic channels.

In one branch we postulate that ADP leaving the enzyme spontaneously with no ATP entering the enzyme in another β cleft. Along this branch for the probability density for spontaneous long-time ADP release $P_D^{spon}(\theta)$ decays according to rate constant $k_D(\theta)$, the ADP ejection rate constant at an angle θ. The system follows this kinetic branch at very low ATP concentrations in what was termed in the past "unisite" kinetics.

The second branch of ADP release in scheme from Fig. 2, described by probability density for $P_D^{fast}(\theta)$, is relevant when the ATP concentration is sufficiently high ($[ATP] > 100nM$). In this case, the binding of ATP in subunit β1 occurs before the ADP had a chance to exit from subunit β3. A fast ADP release is induced by the binding ATP, effectively leading to an "exchange of nucleotides" in the F1-ATPase as seen in fig. 3. For a stronger binding-release cooperation, we postulate that the ADP release rate tends to the binding rate in the β neighbor subunit in the synthesis direction, a condition expressed as

$$k_D(\theta + 240°) = k_T(\theta). \quad (S4)$$

Then, $P_E(\theta)$ and $P_D^{fast}(\theta + 240^o)$ obey the same first-order differential equation, Eq. S3. For the same initial condition at $\theta = \theta_0$, i.e., $P_E(\theta_0) = P_D^{fast}(\theta_0 + 240^o) = 1$, their solutions are identical, except for a 240° shift. For a sufficiently slow rotation rate ω an ADP will be released in all cycles and so, for the "long binding" in Fig. 2.A,

$$P_E(\theta) = P_D^{fast}(\theta + 240^o). \quad (S5)$$

The identity in Eq. S5 was confirmed in single-molecule rotation experiments in which ATP binding is seen to occur in concert with ADP release in the neighboring β subunit in the hydrolysis direction.

We consider now the scenario that both kinetic branches present, one of spontaneous ADP release (unaided by the binding of ATP) and one of fast ADP release. Then, the equation for the ADP release rate is a sum of the spontaneous and fast populations, $P_D = P_D^{spon} + P_D^{fast}$, yielding for the total population,

$$-\omega \frac{dP_D(\theta + 240^o)}{d\theta} = [k_D(\theta + 240^o) + k_T(\theta)]\, P_D(\theta + 240^o). \quad (S6)$$

Since the second term is proportional to the ATP concentration, at high ATP concentration it will be the dominant term and so Eq. S6 will become asymptotically identical to Eq. S3. Conversely, in the limit of low ATP concentrations the first term will be the dominant term. Solving Eqs. S3 and S6 requires the knowledge of the angle-dependent rate constants $k_D(\theta)$ and $k_T(\theta)$, given in the main text, but also in the following paragraphs. The ATP binding rate constant is assumed to be written in terms of a bimolecular rate constant k_{T0}, the ATP concentration [ATP] and an angle-dependent term $g(\theta - \theta_0)$,

$$k_T(\theta) = k_{T0}[ATP]g(\theta - \theta_0). \quad (S7)$$

The function $g(\theta - \theta_0)$ has a 'volcano' shape, with a turnover and exponential slopes, as seen in Fig. 3. When the rate of rotation ω is sufficiently low, the ATP is bound before the turnover seen in the angle-dependent rate constants, i.e., while the θ angle is on the left side of the 'volcano'. So, in this monotonous range the angle-dependence can be approximated with an exponential function, $g(\theta - \theta_0) \approx e^{a(\theta-\theta_0)}$. This exponential dependence on the angle was observed experimentally [7] and treated theoretically using a model of molecular transfer [8]. The angle θ_0 is a reference angle and its value is taken for convenience at the onset of binding during rotation. A typical choice is $\theta_0 = -50°$ where the binding rate is negligible, i.e., is much smaller than the rotation rate ω. This assignment is based upon the experimental rate constants from Fig. 4.A and is aimed to ensure that effectively no binding event registered at θ_0.

We postulate that the angle dependent profile of ADP release rate constant k_D is the same as that of ATP binding, but shifted by $240°$. This relationship, confirmed in the rate constants extracted from the single-molecule experiments in Fig. 4. Formally, the release rate constant is written as

$$k_D(\theta + 240°) = k_{D0}\, g(\theta - \theta_0), \quad (S8)$$

and a consequence is that at the onset of release at $\theta_0 + 240° \approx 190°$ the rate of release is negligible. Using this unified angle-dependent form then, similar formal solution listed as Eqs. 10 and 11 in the main text,

$$ln\, P_E(\theta) \;=\; -\frac{k_{T0}[ATP]}{\omega}\int_{\theta_0}^{\theta} g(\theta' - \theta_0)d\theta'. \tag{S9}$$

$$\ln P_D(\theta + 240°) \;=\; -\frac{k_{D0} + \; k_{T0}[ATP]}{\omega}\int_{\theta_0}^{\theta} g(\theta' - \theta_0)d\theta'. \tag{S10}$$
